# Sodium thiosulfate and pamidronate for treatment of calciphylaxis: case report

**DOI:** 10.25100/cm.v49i3.4134

**Published:** 2018-12-30

**Authors:** Sonia Fernández Cañabate, Cristina Lucas Alvarez, Luis Ortega Valin, Jorge Estifan Ksabji

**Affiliations:** 1 Complejo Asistencial Universitario de León, Servicio de Farmacia, León, España; 2 Complejo Asistencial Universitario de León, Servicio de Nefrología, León, España

**Keywords:** Calciphylaxis, sodium thiosulfate, pamidronate, Hyperphosphatemia, Arterioles, Hypoalbuminemia, Hyperparathyroidism, Calcifilaxis, tiosulfato sódico, pamidronato, hiperfosfatemia, arteriolas, hipoalbuminemia, hiperparatiroidismo

## Abstract

**Introduction:**

Calciphylaxis is an infrequent disease that almost exclusively affects patients with chronic kidney disease, although cases have been observed in patients without renal function impairment. The diagnosis is mainly made by clinical manifestations and subsequently confirmed by radiological and histological study. The optimal treatment is not known, although there is a consensus that a multifactorial approach is required.

**Clinical Case:**

A 68-year-old woman on hemodialysis for 2 years, who presented a painful nodular lesion in the left thigh, a skin biopsy was performed resulting in a diagnosis of calciphylaxis.

**Treatment and Outcome:**

Treatment was started with intravenous sodium thiosulfate. Pamidronate is added intravenously, three months later, due to an unfavorable evolution. After 6 months of treatment, improvement in nodular lesions and healing of the ulcerated lesion was observed to be generally well tolerated treatment.

**Conclusion:**

The combined treatment of sodium thiosulfate, pamidronate and calcitomimetics has been effectiveand safe for the treatment of calciphylaxis, inducing complete remission.

## **Resumen**

**Introducción:**

La calcifilaxis es una enfermedad infrecuente que afecta casi exclusivamente a pacientes con insuficiencia renal, aunque se han observado casos en pacientes sin deterioro de la función renal. El diagnóstico es clínico confirmándose con estudio radiológico e histológico. No se conoce con exactitud el tratamiento óptimo, aunque hay consenso en que se requiere un abordaje multifactorial.

**Caso Clínico:**

Mujer de 68 años en hemodiálisis desde hace 2 años, que presenta una lesión nodular dolorosa en muslo izquierdo, resultando un diagnostico compatible con calcifilaxis, tras biopsia cutánea.

**Tratamiento y resultado:**

Inicia tratamiento con tiosulfato de sodio vía venosa. Tres meses más tarde y ante la evolución desfavorable, se añade al tratamiento pamidronato vía intravenosa. Tras 6 meses de tratamiento se observa mejoría de las lesiones nodulares y cicatrización de la lesión ulcerada, habiéndose experimentado buena tolerancia.

**Conclusión:**

El tratamiento combinado de tiosulfato de sodio, pamidronato y calcimiméticos ha resultado efectivo y seguro para el tratamiento de la calcifilaxis, induciendo su remisión completa.

## Introduction

Calciphylaxis or calcific uremic arteriolopathy (CUA) is a rare and severe disorder characterized by systemic medial calcification of arteries and arterioles that leads to skin and soft tissues ischemic necrosis with the development of ulcers ^1^.

It occurs primarily in patients who have end-stage renal disease (ESRD) and hemodialysis treatment. The incidence has been reported to be up to 4% among these patients. However, it may also occur in non-ESRD patients ^2^.

The exact pathogenesis of CUA is unclear so it is likely multifactorial. Several risk factors are known: hyperparathyroidism, hyperphosphatemia, calciums salts, high doses of vitamin D analogs. It has also been related to obesity, female sex, diabetes, hypoalbuminemia and hypercoagulable states due to protein C and S deficiency or coumarin type anticoagulant therapy ^1^ ^,^ ^2^.

There is no agreement on optimal treatment. Therapeutic must be multifactorial approach and aimed at controlling plasma calcium and phosphate concentrations: ending or control of risk factors associated, avoidance of local tissue trauma and careful local ulcers care and pain control of those wound associated ^3^.

Our objective is to report an infrequent case of CUA treated with sodium thiosulfate and pamidronate and showed good results.

## Case report

A 68-year-old woman, no known drug allergies; with ESRD stage 5D secondary to chronic glomerulonephritis on hemodialysis in later 3 years. She had medical history of hypertension; a history of morbid obesity (body mass index [BMI]= 41.5), she underwent gastric bypass 13 years ago (current weight 56 ​​Kg), type 2 diabetes mellitus, primary hypothyroidism, secondary hyperparathyroidism and mitral insufficiency. She was an ex-smoker, stopping smoking 18 years ago, without any known toxic habits. She suffered a pulmonary thromboembolism 22 years ago, which was treated with acenocoumarol. In addition, the patient receives treatment with sevelamer, paricalcitol, cinacalcet, epoetin alfa, levothyroxine, folic acid, antidiabetic treatment and omeprazole.

**Figure 1:**
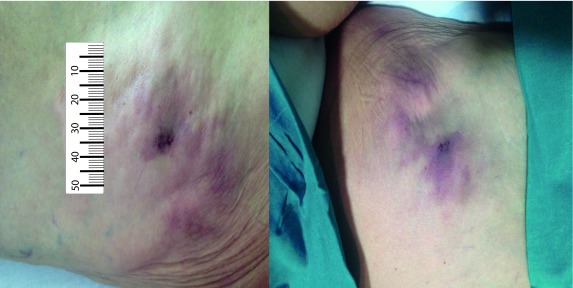
Subcutaneous, non-ulcerated and violaceous nodules on her left front and back thigh respectively, prior to the treatment.

In May 2017, a painful nodule on her left posterior thigh was observed, which was diagnosed as a lipoma by ultrasound. Two months later, the patient reported a significant increase in pain and the nodule size and between 3 and 4 new nodules appeared on her left front and back thigh (Fig. 1). These nodules are firm, adherent and painful on palpation.

**Figure 2:**
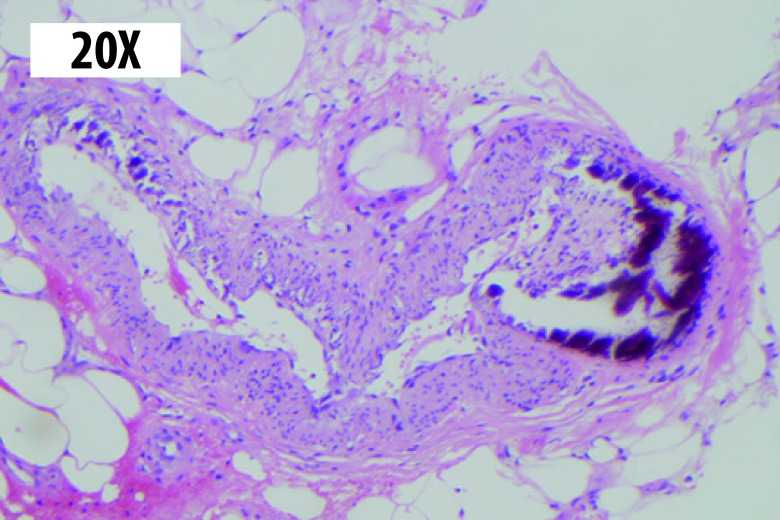
Skin biopsy: hematoxylin-eosin stain 20x. Dystrophic calcification in medium size arteries with intimal proliferation is observed at the hypodermis level.

Dermatology Service was interconsulted, and a skin biopsy was performed obtaining anatomopathological findings compatible with calciphylaxis (Fig. 2).

On a chest and abdominal radiography showed vascular calcifications of arteries.

In view of calciphylaxis diagnosis and by ruling out other disorders that may mimic CUA, the following measures were taken:

- Acenocoumarol therapy was discontinued and instead low molecular weight heparin therapy was started, still maintained today.
- VDRA (Vitamin D Receptor Activator -paricalcitol) was also discontinued, maintaining the rest of treatment.
- Metabolic acidosis was corrected, vitamin k plasma levels were determined, and dialysis bath calcium was lowered.
- The administration of intravenous sodium thiosulfate 12.5 g after each hemodialysis session was started simultaneously as calciphylaxis treatment.

After the skin biopsy a deep ulcer without granulation tissue (1.0 to 1.5 cm diameter) was formed, with no infection which resolved after 5 months of cures and usual follow-up satisfactorily.

Three months after diagnosis, due to an unfavorable evolution and the increase in plasma calcium levels, a bisphosphonate therapy was started (intravenous pamidronate 30 mg weekly) because of its ability to inhibit vascular calcification and its anti-inflammatory effect.

Due to poor digestive tolerance, cinacalcet was replaced by etelcalcetide at an ascending dose of 5 mg intravenously injection after each hemodialysis session due to its lower incidence of gastrointestinal effects.

Three weeks later, because of the marked decrease in the serum calcium and phosphorous concentrations, we occasionally added a treatment with paricalcitol 1.0 mcg after hemodialysis, thus controlling the elevated levels of parathyroid hormone (iPTH).

**Figure 3:**
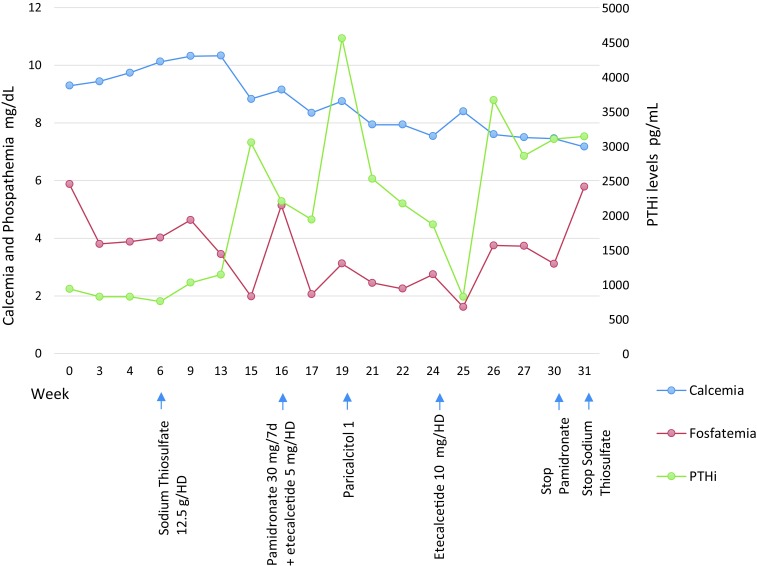
Evolution of analysis of serum biochemical parameters at the beginning and end of each therapy. The average serum levels ​​of calcium and phosphate were 8.66 mg/dL (8.2-10.2 mg/dL] and 3.52 mg/dL (2.7-4.5 mg/dL) respectively, with and average calcium x phosphate product of 30.78 mg/dL. The average value of PTHi was 2,075.89 pg/mL (15-65 pg/mL).

Two months after initiating treatment with etelcalcetide, the dose was increased to 10 mg after each hemodialysis session due to lack of control in iPTH levels and one month later treatment with Pamidronate was stopped due to the appearance of severe maintained hypocalcemia. (Ca = 7.5 mg / dL) (Fig. 3).

Finally, after 6 months of treatment, sodium thiosulfate was suspended due to favorable evolution of the wounds and pain reduction.

The patient currently continues on hemodialysis, stable, without painful lesions and with size reduction of the nodules, making it necessary to increase the dosage of etelcalcetide to 15 mg 3 times a week due to poor control and maintenance high levels of iPTH (Fig. 3).

### Informed consent 

Written informed consent was obtained from the patient for the publication of this case report. 

## Discussion

The CUA is an infrequent disease that almost exclusively affects patients with renal impairment although any cases had been reported in patients without renal disease.^1^. 

The diagnosis is mainly made by clínical manifestations (painful ulcerated lesions) and is confirmed by radiological and histological studies. ^3^. In this case report, our patient had extremely painful violaceus subcutaneous nodules. In general, these lesions progress in a few days to necrotic ulcerations covered with black eschars. However, in our patient, the only deep ulcer observed was due to skin biopsy (in right lower extremity). Although skin biopsy is considered the gold standard for calciphylaxis diagnosis, its realization is controversial due to the high risk of ulceration and subsequent infection. 

Vedvyas *et al*. ^4^, in their review of CUA treatment established that the main objective was the control of risk factors, wound care and pain treatment, emphasizing that a multidisciplinary approach is often required.

Our patient presented several risk factors (ESRD, female gender, history of obesity, diabetes mellitus, hyperparathyroidism, hyperphosphatemia and treatment with acenocoumarol and vitamin D receptor activator), so that we acted on those that could be modified pharmacologically. Specifically, the electrolyte imbalances were addressed, continuing with the use of non-calcium-containing phosphate binders, such as sevelamer; as well stopping treatment with acenocoumarol that it may contribute to disease´s progression.

Treatment with selective vitamin D receptor activator (paricalcitol) was discontinued, as it may increase to development of calciphylaxis indirectly through its action to increase serum calcium and serum phosphate and which can lead to intravascular calcium deposits in arterioles and venules ^2^. However, because of severe and maintained hyperparathyroidism and after control of calcium levels, its reintroduction was necessary.

The control of secondary hyperparathyroidism can be performed using calcimimetics (Allosteric calcium-sensing receptor agonist in parathyroid cells, lowering the secretion of iPTH) or by surgical parathyroidectomy in the absence of response to calcimimetics ^5^. High iPTH levels are a risk factor for CUA ^6^. Our patient maintained high iPTH levels so that surgical parathyroidectomy was the primary treatment, however we decided to be postponed it due to improvement in calciphylaxis lesions and the patient's refusal to undergo surgery.

Bisphosphonates have been shown to be potent inhibitors of osteoclast activity and bone resorption and they are used extensively to the treatment of osteoporosis, cancer-induced hipercalcemia and Paget's disease, and cases have been described in which their use in the treatment of CUA has obtained a good response ^7^ ^,^ ^8^. Due to its renal toxicity, its use is contraindicated in patients with ESRD, however given the severity of the symptoms, the potential benefit of reducing CUA mortality was prioritized. The choice of intravenous bisphosphonate was based on greater experience according to the references, and on the suspicion of a low therapeutic adherence by the patient. However, the induced hypocalcemia motivated its early withdrawal.

Sodium thiosulfate is a drug with proven efficacy and available as a Pharmaceutical compounding. The mechanism of action is unknown, although it is related to its ability to dissolve calcium deposits within tissues through the formation of soluble calcium thiosulfate complexes, and also due to its antioxidant action to improve endothelial dysfunction ^4^. Its administration can cause nausea, vomiting, metabolic acidosis or hypotension in case of rapid infusion. The optimal dose has not been established, however the most reported dose for hemodialysis patients is 25 g 3 times per week after each hemodialysis session, reducing the dose to 12.5 grams for those patients who weigh less than 60 Kg, in order to decrease the incidence of side effects ^1^. The choice of dose in our patient was based on their weight (56 Kg.) without observing adverse effects associated with its administration.

Combination therapy with sodium thiosulfate and bisphosphonates is an uncommon treatment strategy and no many published literature ^9^ ^,^ ^10^; only one case of calciphylaxis unsuccessfully treated with the combination of intravenous thiosulfate and pamidronate has been reported ^11^. However, due to the patient's lack of control of calcium levels (a factor that may contribute to the development of calciphylaxis) and the worsening of the lesions, we decided to use them simultaneously in order to reduce vascular calcification.

## Conclusion 

Sodium thiosulfate in combination with pamidronate and calcimimetics for treatment of CUA, has been effective and relatively safe, giving rise to the clinical remission with absence of pain and drastic size reduction of the nodules with disappearance of any of them, in a disease with poor prognosis.
