# Supplementary material for: Sodium thiosulfate and pamidronate for treatment of calciphylaxis: case report
Source: Colomb Med (Cali). 2018 Dec 30;49(4):288–91. doi: 10.25100/cm.v49i3.4134 (PMC6342083; doi:10.25100/cm.v49i3.4134)
Supplement: Related texts

# Case report: Tiosulfato de sodio y pamidronato para el tratamiento de calcifilaxis: reporte de caso

## Introducción

La calcifilaxis o arteriolopatía urémica calcificante (CUA) es un trastorno raro y grave que se caracteriza por la calcificación de la capa media de las arterias y arteriolas que da lugar al desarrollo de necrosis isquémica de la piel y de los tejidos blandos con formación de úlceras ^1^.

Afecta con mayor frecuencia a pacientes que presentan una enfermedad renal crónica (ERC) y en diálisis. Entre estos pacientes se alcanza una prevalencia de hasta el 4%. Sin embargo, también puede afectar a pacientes que no padecen ERC ^2^.

No está bien establecida la patogenia de la CUA por lo que es probable que sea multifactorial. Se conocen varios factores de riesgo: hiperparatiroidismo, hiperfosfatemia, análogos del calcio, altas dosis de metabolitos de la vitamina D. También se ha relacionado con la obesidad, el sexo femenino, la diabetes, la hipoalbuminemia y con estados de hipercoagulabilidad por deficiencia de las proteínas C y S o con el uso de anticoagulantes orales tipo cumarínicos ^1^^,^^2^.

No existe un acuerdo sobre el tratamiento óptimo. El abordaje terapéutico debe ser multifactorial y estar dirigido a la normalización de los niveles del calcio y el fósforo: la supresión o control de los factores de riesgo que están asociados, evitar los traumatismos y realizar un cuidado intensivo de las ulceras y un control del dolor asociado a dichas lesiones ^3^.

Nuestro objetivo es presentar un caso infrecuente de CUA tratado con tiosulfato y pamidronato que arrojó buenos resultados.

## Descripción del caso

Mujer de 68 años de edad, sin alergias medicamentosas conocidas; con ERC estadío 5D secundaria a glomerulonefritis crónica en hemodiálisis de 3 años de evolución. Presentó hipertensión arterial; con antecedentes de obesidad mórbida (IMC 41.5), fue sometida a bypass gástrico hace 13 años (peso actual de 56 Kg), presenta diabetes mellitus tipo II, hipotiroidismo primario, hiperparatiroidismo secundario e insuficiencia mitral. Exfumadora, abandonando el hábito tabáquico hace 18 años, sin presentar ningún otro hábito tóxico. Hace 22 años sufrió un tromboembolismo pulmonar, que fue tratado con acenocumarol. Además la paciente está en tratamiento con sevelamero, paricalcitol, cinacalcet, epoetina alfa, levotiroxina, ácido fólico, tratamiento antidiabético y omeprazol.

En mayo de 2017 se observó la aparición de un nódulo doloroso en la cara posterior del muslo izquierdo, que fue diagnosticado como lipoma mediante ecografía. Dos meses más tarde, la paciente refirió un aumento considerable del dolor y del tamaño del nódulo y aparecieron entre 3-4 nuevos nódulos en la cara anterior y posterior del muslo izquierdo (Fig. 1). A la palpación estos nódulos son firmes, adheridos y dolorosos.

**Figura 1:** Nódulos subcutáneos, no ulcerados y violáceos en región anterior y posterior del muslo izquierdo respectivamente, previo al inicio del tratamiento.

Posteriormente se realizó una interconsulta con el Servicio de Dermatología donde le practicaron una biopsia de la lesión. El diagnóstico anatomopatológico fue compatible con calcifilaxis (Fig. 2).

**Figura 2:** Biopsia cutánea: tinción de hematoxilina-eosina 20x. Se aprecia, a nivel de hipodermis, calcificación distrófica de la pared de vasos de mediano calibre con proliferación intimal.

En la radiografía simple de tórax y abdomen se observaron calcificaciones vasculares en trayectos arteriales.

Ante el diagnóstico de calcifilaxis y tras descartar otros diagnósticos de calcinosis, se tomaron las siguientes medidas:

- Se suspende el tratamiento con acenocumarol y en su lugar se inició un tratamiento con heparina de bajo peso molecular manteniéndose en la actualidad.
- La vitamina D (paricalcitol) también se suspendió, manteniéndose el resto de la medicación.
- Se corrigió la acidosis metabólica, se determinaron los niveles de vitamina K, y se redujeron las concentraciones de calcio en el baño de diálisis.
- Se inició simultáneamente la administración de 12.5 g de tiosulfato sódico intravenoso tras cada sesión de hemodiálisis como tratamiento de la calcifilaxis.

Tras la biopsia cutánea se formó una úlcera profunda de 1.0-1.5 cm pero sin tejido de granulación, no manifestándose ninguna infección y resolviéndose satisfactoriamente tras 5 meses de curas y seguimientos habituales. 

Tres meses después del diagnóstico, tras evidenciar empeoramiento clínico y considerando el aumento de la calcemia, se comenzó un tratamiento con bifosfonato (pamidronato intravenoso a dosis de 30 mg semanal) por su capacidad de inhibir la calcificación vascular y su efecto antiinflamatorio. 

Ante la persistencia de la mala tolerancia digestiva, se sustituyó el cinacalcet por la etelcalcetida a dosis ascendente de 5 mg intravenoso directo después de cada sesión de hemodiálisis debido a su menor incidencia de efectos gastrointestinales.

Tres semanas más tarde, ante el descenso marcado de la calcemia y de la fosfatemia añadimos de modo ocasional un tratamiento con paricalcitol 1.0 mcg post hemodiálisis controlando así los niveles elevados de la hormona paratiroidea (PTHi).

Dos meses después de iniciar el tratamiento con etelcalcetida se incrementó la dosis a 10 mg después de cada sesión de hemodiálisis ante la falta de control en los niveles de la PTHi y un mes más tarde se suspendió el tratamiento con Pamidronato por la aparición de una hipocalcemia severa mantenida (Ca= 7.5 mg/dL) (Fig. 3).

**Figura 3:** Evolución de los principales parámetros bioquímicos analizados y el inicio y finalización de cada terapia utilizada. Los valores medios de calcio y fósforo fueron de 8.66 mg/dL (8.2-10.2 mg/dL) y 3.52 mg/dL (2.7-4.5 mg/dL) respectivamente con un producto calcio-fósforo medio de 30.78 mg/dL. El valor medio de PTHi fue de 2,075.89 pg/mL (15-65 pg/mL).

Finalmente, tras 6 meses de tratamiento, se suspendió el tiosulfato sódico por la evolución favorable de las lesiones y disminución del dolor. 

Actualmente la paciente continua en hemodiálisis, estable, sin lesiones dolorosas y con disminución del tamaño de los nódulos, haciéndose necesario el aumento de las dosis de la terapia con etelcalcetida a 15 mg 3 veces por semana debido al mal control y al mantenimiento de unos niveles de PTHi elevados (Fig. 3). 

### Consentimiento informado

Se obtuvo el consentimiento informado por escrito de la paciente para la publicación de este caso.

## Discusión

La CUA es una enfermedad infrecuente que afecta casi exclusivamente a pacientes con insuficiencia renal, aunque se han observado casos en pacientes sin esta afectación ^1^. 

El diagnóstico es clínico (dolor, presencia de lesiones ulceradas dolorosas, etc.) confirmándose con el estudio radiológico e histológico ^3^. En el caso que presentamos, se manifestaban una serie de nódulos subcutáneos, violáceos, extremadamente dolorosos. De manera general estas lesiones progresan en pocos días a la formación de úlceras necróticas recubiertas de escaras negruzcas. No obstante, en nuestro caso la única úlcera profunda que se observó fue secundaria a la biopsia cutánea en la extremidad inferior derecha. A pesar de que es la prueba de referencia para el diagnóstico de calcifilaxis, su realización está cuestionada por el riesgo de ulceración y posterior infección. 

Vedvyas *et al*. ^4^, en su revisión del tratamiento de la CUA establecieron que el objetivo era el control de los factores de riesgo, el cuidado de las heridas y el tratamiento del dolor, resaltando que a menudo se requiere de un abordaje multidisciplinar. 

Nuestra paciente presentaba numerosos factores de riesgo (ERC, sexo femenino, antecedentes de obesidad, diabetes mellitus, hiperparatiroidismo, hiperfosfatemia y tratamiento con acenocumarol y derivados de la vitamina D), por lo que se actuó sobre aquellos que podían ser modificados farmacológicamente. En concreto, se abordaron los desequilibrios electrolíticos continuando con el uso de quelantes de fósforo que no aportan calcio, como es el sevelámero; así como la supresión de tratamiento con acenocumarol ya que se ha comprobado que favorece la progresión de la enfermedad.

Se suspendió el tratamiento con el análogo de vitamina D (paricalcitol), ya que puede contribuir al desarrollo de calcifilaxis por la hipercalcemia e hiperfosforemia que puede producir y que puede dar lugar a depósitos de calcio intravascular en arteriolas y vénulas ^2^. Sin embargo debido al hiperparatiroidismo severo y mantenido de la paciente y tras control de los niveles de calcio, fue necesario su reintroducción. 

El control del hiperparatiroidismo secundario se realizó mediante el empleo de calcimiméticos (agonistas alostéricos de los receptores de calcio en las células paratiroideas reduciendo así la secreción de PTHi) o bien mediante la extirpación de las paratiroides ante la falta de respuesta a los calcimiméticos ^5^. Los altos niveles de PTHi son un factor de riesgo de la CUA ^6^. Nuestra paciente mantuvo cifras elevadas de PTHi por lo que el tratamiento terapéutico indicaba paratiroidectomía quirúrgica, sin embargo la mejora en las lesiones de la calcifilaxis y ante la negativa de la paciente a someterse a una intervención quirúrgica se decidió posponer.

Los bisfosfonatos tienen un potente efecto inhibidor sobre la actividad de los osteoclastos y la resorción ósea y se utilizan en gran medida en el tratamiento de la osteoporosis, la hipercalcemia tumoral y la enfermedad de Paget, además se han descrito casos en los que su empleo en el tratamiento de la CUA ha obtenido una buena respuesta ^7^^,^^8^. Debido a su toxicidad renal, su uso en pacientes con ERC está contraindicado, sin embargo dada la gravedad de los síntomas, se priorizó el potencial beneficio de reducir la mortalidad de la CUA. La elección del bifosfonato intravenoso se basó en una mayor experiencia según la bibliografía consultada, y en la sospecha de baja adherencia por parte de la paciente. No obstante la hipocalcemia provocada motivó su retirada temprana.

El tiosulfato sódico es un fármaco con eficacia demostrada y disponible como fórmula magistral. Se desconoce el mecanismo de acción, aunque se relaciona con su capacidad de disolver los depósitos de calcio en los tejidos mediante la formación de complejos solubles, y por su acción antioxidante mejorando la disfunción endotelial ^4^. Su administración puede dar lugar a nauseas, vómitos, acidosis metabólica o hipotensión en caso de una infusión rápida. No se ha establecido la dosis óptima, sin embargo la dosis más reportada para los pacientes en hemodiálisis es de 25 g tres veces por semana después de la sesión, reduciéndose a 12.5 g para aquellos pacientes con peso inferior a 60 Kg, con el fin de disminuir la incidencia de efectos adversos ^1^. La elección de la dosis en la paciente fue con base en su peso (56 Kg.) sin observarse efectos adversos asociados a su administración. 

El uso de una terapia combinada de tiosulfato de sodio y bifosfonato es una estrategia poco utilizada y con poca bibliografía publicada ^9^^,^^10^; solamente se ha descrito un caso de calcifilaxis tratada sin éxito con la combinación de tiosulfato intravenoso y pamidronato ^11^. Sin embargo debido a la falta de control de los niveles de calcio (factor que puede contribuir al desarrollo de calcifilaxis) en la paciente y al empeoramiento de las lesiones se decidió su utilización simultánea con el fin de reducir las calcificaciones vasculares. 

## Conclusión 

El uso combinado de tiosulfato, pamidronato y calcimiméticos para el tratamiento de CUA ha resultado efectivo y relativamente seguro, dando lugar a la remisión del cuadro con ausencia de dolor y disminución drástica del tamaño de los nódulos con desaparición de alguno de ellos, en una enfermedad de mal pronóstico.
